# Spatiotemporal clustering, socioeconomic drivers and policy evaluation of mumps in Hunan Province, China, 2015–2024

**DOI:** 10.1016/j.pmedr.2026.103601

**Published:** 2026-08-17

**Authors:** Linlong Liang, Xiaoyan Liu, Fuqiang Liu, Lin Liu

**Affiliations:** a Department of Pediatrics, the Affiliated Changsha Central Hospital, Hengyang Medical School, University of South China, Changsha, China; b Science, Education and International Cooperation Department, Hunan Provincial Center for Disease Control and Prevention, Changsha, China

**Keywords:** Mumps, Spatiotemporal clustering, Geographically and temporally weighted regression, Interrupted time series

## Abstract

**Objective:**

To analyze the spatiotemporal clustering of mumps in Hunan Province from 2015 to 2024 and its influencing factors and to explore the policy effect of adjusting the immunization schedule.

**Methods:**

Spatial autocorrelation, spatiotemporal clustering, geographically and temporally weighted regression (GTWR), and interrupted time series (ITS) analyses were performed on county-level mumps surveillance data obtained from Hunan Province for 2015–2024 to identify mumps clustering, quantify socioeconomic drivers and assess the two-dose mumps-containing vaccine (MuCV) policy impact.

**Results:**

In total 157,502 cases were reported, and 89.7% of patients were aged 0–14 years. The global Moran's *I* increased from 0.20 to 0.67 (*P* < 0.01), indicating intensified clustering. Despite the decreasing provincial incidence, high-high hotspot geographic coverage expanded because of widening spatial disparities. The primary hotspot was in northeastern Hunan (Nov 2016–Dec 2019). GTWR revealed that per capita gross domestic product (GDP) and population mobility intensity had positive local effects in northeastern counties (adjusted *R*^*2*^ = 0.45). ITS showed an immediate significant decline after the 2020 policy (*β*_*2*_ = −2.50, *P* < 0.01).

**Conclusions:**

Persistent hotspots in northeastern Hunan may be driven by per capita GDP and intercounty mobility. The two-dose policy reduced overall incidence but exacerbated regional inequalities.

## Highlights

• Mumps clustering increased in Hunan from 2015 to 2024 despite the lower incidence.
• A hotspot persisted in northeastern Hunan from November 2016 to December 2019.
• Per capita economic output and mobility drove mumps transmission in the northeast.
• Two-dose mumps vaccination decreased the incidence but widened the disparities.
• Targeted vaccination and screening within hotspots are recommended.

## 1 Introduction

Mumps is an acute respiratory infectious disease caused by the mumps virus, and is spread primarily through respiratory droplets and direct contact (Hviid et al., 2008; Noor and Krilov, 2023). The usual clinical feature is painful swelling of one or both parotid glands, often with systemic symptoms such as fever, headache, and a sense of malaise (Sun et al., 2020). In some cases, mumps may lead to severe complications, including orchitis, oophoritis, and viral encephalitis (Latner and Hickman, 2015; Wu et al., 2021).

The use of MuCV is the most cost-effective control measure (van Druten et al., 1986; Xiao et al., 2026). High two-dose coverage reduces the incidence to less than 1/100,000, yet outbreaks persist in countries with high coverage, such as the US (Shepersky et al., 2021) and Canada (Saboui and Squires, 2020), because of waning immunity (Demicheli et al., 2012; Di Pietrantonj et al., 2021). These findings indicate that mumps control remains an ongoing public health challenge.

In 2008, China incorporated single-dose MuCV into its National Immunization Program (NIP) to address the ongoing issue of mumps (Lin et al., 2021). As a Category I vaccine under the NIP, the MuCV is provided free of charge to all eligible children and is administered in accordance with mandatory national immunization regulations. In June 2020, the immunization schedule was revised to a two-dose regimen, with doses administered at 8 months and 18 months of age. Official notification data show that from 2020 to 2024, the coverage rate of the MuCV in Hunan Province remained above 90%. Serological surveys revealed significantly greater antibody positivity (98.6%) and geometric mean concentration (928.5 IU/mL) in two-dose recipients than in one-dose recipients. Nevertheless, the incidence in Hunan still exceeds the national average, indicating persistent transmission.

Current studies on mumps in Hunan primarily report conventional epidemiology but lack spatial analyses of transmission dynamics and clustering. To address this gap, we systematically assessed the spatiotemporal distribution and clustering of mumps (2015–2024) using GTWR and interrupted time series to quantify socioeconomic drivers and the two-dose vaccine policy effect.

## 2 Methods

### 2.1 Study design and population

Hunan Province is located in south-central China (108°47′–114°15′ E, 24°38′–30°08′ N). The province features a distinctive topographical configuration with mountain ranges bordering three sides and forming a horseshoe-shaped basin that opens toward the north; it is subject to a typical subtropical monsoon climate. As of the end of 2024, Hunan covered a total administrative area of 211,800 km^2^ and had a permanent resident population of 65.4 million. The provincial administrative structure comprises 14 prefecture-level divisions and 122 county-level subdivisions. All these geographical and administrative units are encompassed within the scope of this study.

### 2.2 Measures

From 2015 to 2024, mumps surveillance data for Hunan Province were collected from the Chinese Information System for Disease Control and Prevention. The diagnosis complies with the “Mumps Diagnostic Criteria” (WS 270–2007), excluding non-mumps cases such as acute suppurative mumps and lymphadenitis, with no missing information. The information gathered included the case number, sex, age, administrative code for the county or urban district, onset date, diagnosis date, and occupation.

All the data were deidentified prior to analysis, and no personal identifiers (e.g., name, address, contact information, medical record numbers) were included. All the data obtained in this study were independently reviewed by two researchers, and duplicate cases were excluded. A vector map of Hunan Province was generated by the National Basic Geographic Information System.

### 2.3 Statistical analysis

#### 2.3.1 Descriptive and geographical analysis

Descriptive statistical techniques were applied to illustrate the epidemiological profile of mumps in Hunan Province from 2015 to 2024. The population included in the analysis was divided into five age groups: 0–4 years, 5–9 years, 10–14 years, 15–19 years, and ≥ 20 years. Moreover, the population was classified into four categories by occupation: scattered children, nursery children, students and others. Thematic maps of the annual incidence rate of mumps at the county level were constructed using ArcGIS® 10.7. The depth of color was used to represent the incidence rate of mumps in each county (Tan et al., 2022).

#### 2.3.2 Spatial autocorrelation analysis

Spatial autocorrelation analysis is categorized into global and local analyses, both of which aim to detect spatial patterns of correlation (Sun et al., 2025; Zhang et al., 2021). The spatial weight matrix for Moran's *I* calculation adopted the Queen contiguity criterion: any two counties sharing a common boundary or vertex were defined as adjacent spatial neighbors. Moran's *I* ranged from −1 to 1, with values >0 indicating positive clustering; a Z score > 1.96 denoted statistical significance at *α* = 0.05 (Mao et al., 2019; Zhao et al., 2025). Moreover, a linear trend regression analysis of the global Moran's *I* from 2015 to 2024 was conducted using SPSS® 18.0 software.

Local indicators of spatial autocorrelation (LISA) (Zhang et al., 2019) cluster maps use distinct colors to represent various types of spatial autocorrelation. LISA was used to categorize spatial patterns into high-high, low-low, high-low, and low-high clusters (Kong et al., 2024; Yang et al., 2021).

#### 2.3.3 Spatiotemporal cluster analysis

Discrete Poisson spatiotemporal scan statistics (SaTScan™ 9.6) were used to identify epidemic clusters, with county-level spatial units and monthly temporal intervals (Kulldorff et al., 2006). The study period spanned from January 2015 to December 2024, and the temporal unit was set at one month. In parallel, in accordance with established methodological practices, the maximum time window was restricted to 50% of the overall study period. The maximum spatial cluster size was tested across 10%, 20%, and 30% of the total provincial population via dedicated sensitivity analysis, and the final 30% spatial window threshold was selected for analysis (Tango and Takahashi, 2005; Yu et al., 2023). Monthly interpolated county population values served as Poisson model population offsets. All 122 county-level analytical units remained geographically consistent from 2015 to 2024. Model significance relied on 999 Monte Carlo replications (Beyene et al., 2018), with the highest log likelihood ratio (*LLR)* clusters defined as primary hotspots (Kulldorff et al., 1997), and secondary clusters ranked by descending *LLR* with *P* < 0.05 (Kleinman et al., 2005).

#### 2.3.4 Geographically and temporally weighted regression analysis

We used GTWR to quantify spatiotemporal nonstationary associations between county-level mumps incidence and five covariates (per capita GDP, population density, density of medical institutions, school density, and population mobility intensity), validated by ordinary least squares (OLS) and geographically weighted regression (GWR). Data were obtained from the Hunan Provincial Statistical Yearbooks and the China County Statistical Yearbooks. Official intercounty population mobility records were unavailable; therefore, population mobility intensity was calculated using a gravity model, in which pairwise county spherical distance represented spatial friction, and mobility volume was proportional to the product of the resident populations of the origin and destination counties and inversely proportional to distance decay. After standardization in SPSS, variance inflation factor (VIF) screening excluded population density (VIF > 10); the remaining four covariates had a VIF < 2. Models were fitted in ArcGIS 10.7; performance was compared using the corrected Akaike information criterion (*AIC*_*C*_), adjusted *R*^*2*^, and residual squares (*RSS)*.

#### 2.3.5 Interrupted time series analysis

We conducted segmented ITS regression to quantify changes in monthly mumps incidence across Hunan Province before and after the two-dose mumps-containing vaccine policy was launched in June 2020. Monthly notifiable mumps surveillance data from January 2015 to December 2024 were extracted, generating a balanced time series dataset with 120 monthly observations.

The segmented ITS regression model was specified as follows:

$${\text{Incidence}}_{t}=\beta_{0}+\beta_{1}{\text{Time}}_{t}+\beta_{2}D_{t}+\beta_{3}\left({\text{Time}}_{t}\times D_{t}\right)+\varepsilon_{t}$$

where Incidence_t_ denotes the monthly mumps incidence rate at month t; Time_t_ is a continuous monthly counter capturing the preintervention baseline trend; D_t_ is a binary dummy (0 before June 2020 and 1 thereafter) that estimates the immediate level shift of incidence; and Time_t_ × D_t_ is the interaction term measuring the postintervention slope change. Newey–West HAC standard errors with lag = 1 were adopted to address serial autocorrelation of monthly infectious disease data. The net long-term trend after policy implementation was calculated as *β*_*1*_+ *β*_*3*_.

#### 2.3.6 Software

Descriptive epidemiological statistics were calculated via SPSS 18.0. Spatial autocorrelation, linear trend regression analysis of the global Moran's *I,* LISA mapping, data standardization, GTWR modeling, and coefficient visualization were performed using ArcGIS 10.7 and SPSS 18.0. Spatiotemporal scan statistics were performed in SaTScan 9.6. Interrupted time series regression and Newey–West standard error estimation were performed using Stata® 18.0. All statistical significance thresholds were set at α = 0.05.

### 2.4 Ethics statement

As the secondary analysis of routine surveillance data in this study fully meets the statutory exemption criteria—being completely anonymized, nonharmful to participants, and free of sensitive personal information or commercial interest involvement—the Ethics Committee of Changsha Central Hospital has confirmed that this study is exempt from ethics approval and formally waived the requirement for individual informed consent.

## 3 Results

### 3.1 Descriptive and geographical analysis

#### 3.1.1 Epidemiological characteristics

In Hunan, from 2015 to 2024, 157,502 mumps cases were reported, with an average annual incidence of 23.4 per 100,000 individuals. Among the patients, 92,904 were male and 64,598 were female, resulting in a male-to-female ratio of 1.44:1. The disease was predominantly observed in children and adolescents aged 0 to 14 years, who accounted for 141,308 cases (89.7% of the total). Within this age group, individuals aged 5 to 14 years accounted for the greatest proportion of cases. Students constituted the most affected subgroup (63.6%), followed by nursery children and scattered children. Mumps exhibited a seasonal pattern, with peak occurrences during summer and winter, which together accounted for 60.9% of all reported cases (Table 1).

**Table 1:** Demographic characteristics of patients with mumps in Hunan Province from 2015 to 2024.

Variables	Case number	Percentage (%)
Sex		
Male	92,904	59.0
Female	64,598	41.0

Age (years)		
0–4	24,432	15.5
5–9	80,302	51.0
10–14	36,574	23.2
15–19	4498	2.9
≥20	11,696	7.4

Occupation		
Scattered children	17,063	10.8
Nursery children	28,872	18.3
Students	100,116	63.6
Other	11,451	7.3

Season		
Spring	28,187	17.9
Summer	55,100	35.0
Autumn	33,381	21.2
Winter	40,834	25.9
Total	157,502	100.0

#### 3.1.2 Spatial incidence distribution

**Fig. 1:**
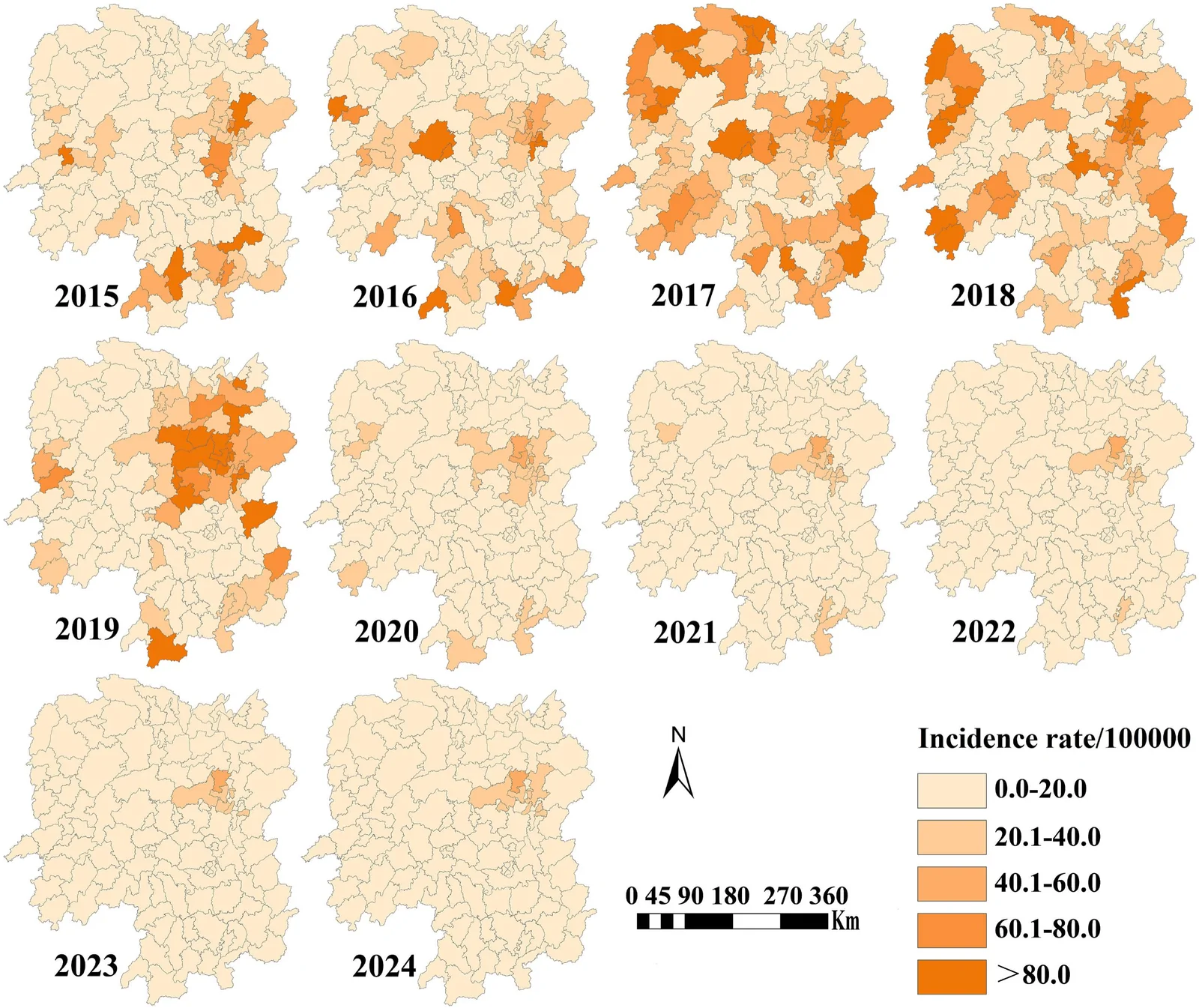
Annual incidence of mumps at the county level in Hunan Province from 2015 to 2024.

The county-level annual mumps incidence in Hunan from 2015 to 2024, averaging 23.4 cases per 100,000 people, is shown in Fig. 1. The incidence increased to a 2017 peak, remained high through 2019, and then declined sharply after 2020 to a low in 2023, with a slight rebound occurring in 2024. High-incidence areas were spatially dispersed in 2017–2018 but became concentrated in northeastern Hunan from 2019 onward, and this northeastern clustering persisted throughout 2020–2024 despite the overall downturn.

### 3.2 Spatial autocorrelation and spatiotemporal clustering patterns

#### 3.2.1 Spatial autocorrelation analysis

**Fig. 2:**
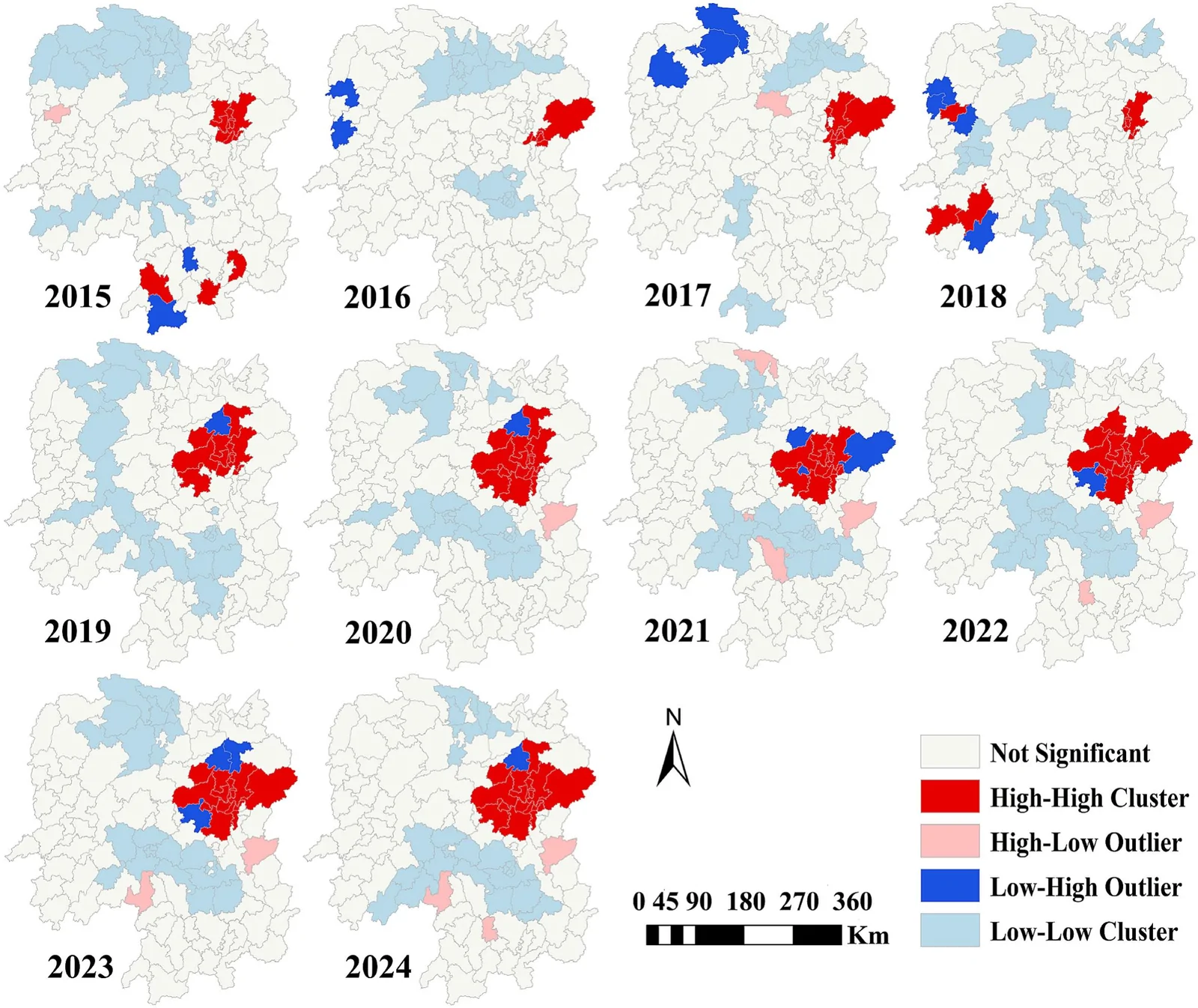
Yearly local indicators of spatial association cluster maps of mumps incidence at the county level in Hunan Province, 2015 to 2024.

A global autocorrelation analysis of mumps incidence in Hunan Province for 2015 to 2024 revealed that the global Moran's *I* indices ranged from 0.20 to 0.67, with all Z values exceeding 1.96 (*P* < 0.01) (Table S1). Linear trend regression analysis revealed that Moran's index increased significantly over time (*B* = 0.05, *P* < 0.01, *R*^*2*^ = 0.75); the Durbin–Watson value was 1.80, and there was no significant autocorrelation in the residuals. These findings suggest a significant positive spatial autocorrelation in mumps incidence at the county level in Hunan Province over the study period, indicating spatial clustering with an increasing trend in aggregation intensity. According to the annual LISA cluster maps, high-high clusters were relatively limited and dispersed between 2015 and 2018 and were primarily concentrated in certain counties and urban districts under the jurisdiction of Changsha and Zhuzhou cities in northeastern Hunan. From 2019 to 2024, the number of counties and urban districts included in high-high clusters increased notably and became more spatially concentrated. The counties and urban districts within Changsha and Zhuzhou continued to represent persistent hotspots, with a particular emphasis on the nine administrative counties and urban districts of Changsha City, which consistently exhibited high-high cluster patterns. Additionally, Xiangxiang City, Shaoshan City, and Xiangtan County under Xiangtan City, as well as Heshan District in Yiyang City, emerged as new or sustained high-incidence agglomeration areas (Fig. 2).

#### 3.2.2 Spatiotemporal cluster analysis

**Fig. 3:**
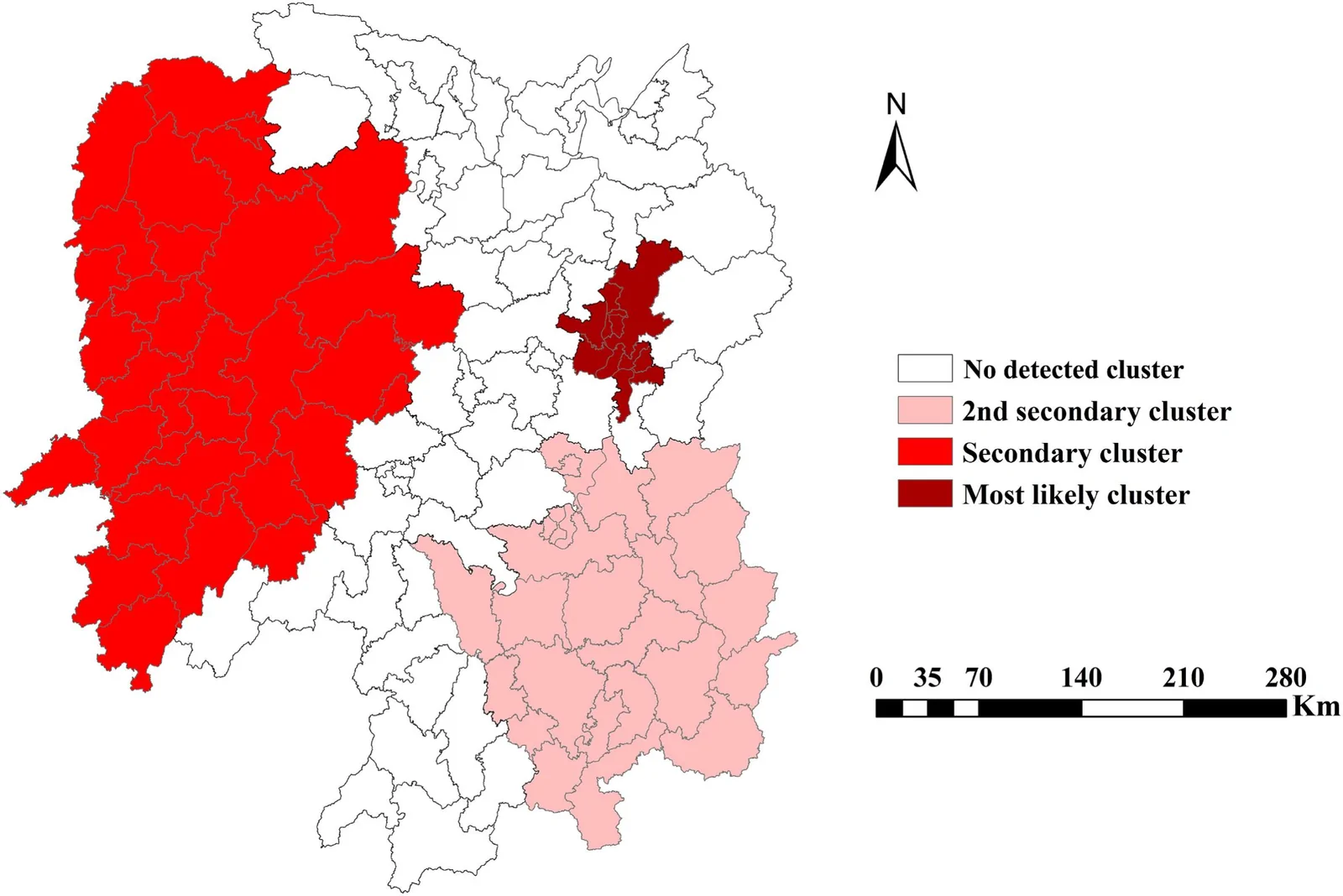
Spatiotemporal clustering of mumps in Hunan Province from 2015 to 2024 (Note: Blank administrative regions indicate areas with no detected clusters.)

We compared the 10%, 20%, and 30% spatial windows (with a fixed 50% time window) through sensitivity analysis. The 10% window caused overfragmentation; the 20% window split contiguous epidemic areas; and the 30% window preserved core cluster robustness (consistent with the 20% window), integrated scattered clusters, and had the greatest *LLR* value (Table S2-S3). Therefore, 30% was selected as the optimal parameter. The results revealed that the main agglomeration area was located in the northeastern part of the province, encompassing Changsha City and Zhuzhou City and included 12 counties and urban districts, such as Kaifu District, Furong District, Yuhu District, and Tianyuan District (Fig. 3). This cluster spanned the period from November 1, 2016, to December 31, 2019, predating the full implementation of the two-dose MuCV vaccination policy. Two secondary clusters were also detected: the first secondary cluster (*n* = 31) was located in the northwestern part of Hunan Province and covered the period from November 1, 2016, to July 31, 2018; the second secondary cluster (*n* = 26) was located in the southeastern region and occurred from April 1, 2017, to July 31, 2018 (Table S4).

### 3.3 Geographically and temporally weighted regression analysis

**Fig. 4:**
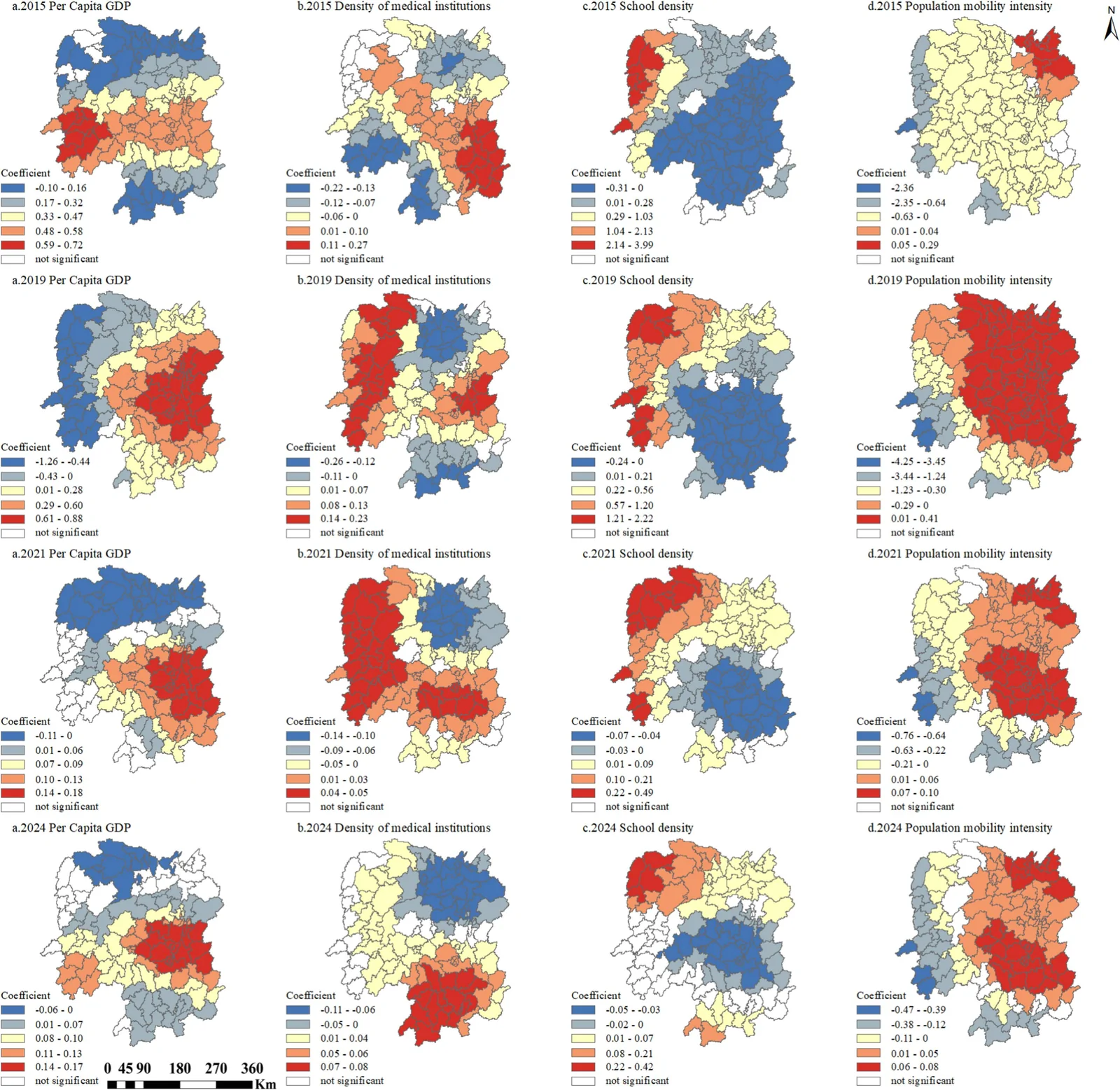
Spatial distribution of geographically and temporally weighted regression coefficients for 2015, 2019, 2021, and 2024.

Compared with OLS and GWR, GTWR achieved a superior fit, with the lowest *AIC*_*C*_ and the highest adjusted *R*^*2*^ (0.45), confirming substantial spatial nonstationarity (Table S5). The per capita GDP (mean = 0.20) gradually exerted stable positive effects in northeastern urban counties. School density (mean = 0.15) displayed sustained, significant positive associations concentrated in northwestern Hunan. Density of medical institutions (mean = 0.02) yielded weak, mostly nonsignificant effects without significant continuous clusters. Population mobility intensity had a provincial average coefficient of −0.18, with positive local effects in northeastern border counties. Collectively, these spatially heterogeneous patterns statistically reconcile the “expanding hotspot with declining provincial incidence” paradox. Nevertheless, the moderate adjusted *R*^*2*^ and the absence of county-level data on potential confounders (e.g., MuCV coverage) limit causal interpretations (Fig. 4, Table S6).

### 3.4 Interrupted time series analysis

A segmented regression of 120 monthly mumps surveillance records (January 2015–December 2024) with Newey–West HAC standard errors revealed a significant baseline incidence (*β*_*0*_ = 2.06, *P* < 0.01). The prepolicy trend was modestly positive but nonsignificant (*β*_*1*_ = 0.02, *P* = 0.10), suggesting that population immunity waned under the single–dose schedule. After the introduction of the two-dose MuCV in June 2020, an immediate substantial reduction (*β*_*2*_ = −2.50, *P* < 0.01) and a significant negative slope change (*β*_*3*_ = −0.03, *P* = 0.05) occurred. The net postintervention trend (*β*_*1*_ + *β*_*3*_ = −0.01, *P* = 0.03) confirmed a sustained long–term decline in monthly incidence through December 2024. Model fit statistics (*LLR* = −194.51, *AIC* = 3.31, and *BIC* = −375.63) indicated acceptable performance. However, concurrent COVID-19 nonpharmaceutical interventions (NPIs) (2020−2022) likely confounded the estimates (Table 2).

**Table 2:** Results of the interrupted time series model for the monthly incidence of mumps in Hunan Province from 2015 to 2024.

Variable	Coefficient	Z value	*P* value
Starting level of mumps incidence (*β*_*0*_)	2.06	5.22	<0.01
Trend for mumps incidence before the intervention (*β*_*1*_)	0.02	1.66	0.01
Level change in mumps incidence following the intervention (*β*_*2*_)	−2.50	−3.91	<0.01
Slope change in mumps incidence following the intervention (*β*_*3*_)	−0.03	−2.01	0.05
Net linear trend for mumps incidence after the intervention	−0.01	−2.20	0.03

## 4 Discussion

We comprehensively applied descriptive epidemiology, spatial autocorrelation, spatiotemporal scanning, GTWR, and ITS to analyze mumps in Hunan (2015–2024), incorporating the two-dose MuCV policy shift. Despite the declining incidence, the distribution showed significant spatial heterogeneity and dynamic clustering. The descriptive findings align with those of other Chinese studies (Li et al., 2022; Peng et al., 2023), highlighting school-aged children (5–14 years) and students as the most affected groups with a bimodal seasonal pattern coinciding with school terms (Yu et al., 2018; Zhu et al., 2019). The higher incidence in males (1.44:1) is consistent with the findings of other reports (Yang et al., 2022) and is potentially associated with broader social contact networks and behavioral factors (van Lunzen and Altfeld, 2014) that may exacerbate transmission in densely populated school settings. The 2020 two-dose policy reduced the overall incidence, and serological data confirmed enhanced immunogenicity. However, >90% coverage notwithstanding, susceptibility persists because of historical gaps, waning immunity, and the incomplete immunization of migrant children (Lewnard and Grad, 2018), warranting booster campaigns and school-health collaboration.

The core findings reveal increasing spatial clustering. The aggregation intensity of mumps incidence at the county level in Hunan Province is increasing (*B* = 0.05, *P* < 0.01, *R*^*2*^ = 0.75). Although the provincial incidence decreased after 2019, high-risk areas expanded, and clustering intensified. To explain this paradox, we integrated Moran's *I* trends and GTWR coefficients. GTWR identified per capita GDP, school density, and population mobility intensity as persistent drivers of mumps transmission. The model further revealed that incidence declined more slowly in hotspots than the provincial average, thereby widening regional disparities while enhancing the detectability of spatial clusters. The persistent hotspot in northeastern Hunan (Changsha–Zhuzhou) covered 12 counties from November 2016 to December 2019 (prepolicy), plus two transient secondary clusters in the northwest and southeast. The formation of the primary clustering areas in northeastern Hunan Province may be related to factors such as per capita GDP and population mobility intensity. The northwest secondary cluster may be related to school density and limited rural vaccination access; the southeast cluster may be related to industrial-driven population inflow. However, the modest adjusted *R*^*2*^ = 0.45 of the GTWR model and missing county-level vaccination data preclude strong causal inference; these interpretations remain tentative.

ITS analysis revealed that the incidence rate decreased significantly immediately after June 2020 (*β₂* = −2.50, *P* < 0.01) and exhibited a long-term continuous downward trend (net slope = −0.01, *P* = 0.03). However, NPIs for COVID-19 implemented during the same period (2020–2022) may have introduced confounding effects. Although the incidence rate declined, high-high spatial clusters expanded simultaneously, suggesting uneven intervention benefits or vaccine coverage gaps. These findings are consistent with the GTWR results: even though the provincial average incidence decreased, per capita GDP and population mobility intensity in northeastern counties and districts continued to drive transmission.

Antibody screening and supplementary vaccination should be prioritized in schools in the continuous hotspots in the northeastern region, and daily monitoring should be strengthened in historical hotspots in the northwestern and southeastern regions. Moreover, collaboration between the education and public health sectors should be enhanced to strengthen school-based infection control measures, including symptom screening, improved ventilation, and case isolation. However, the absence of county-level vaccination data and the moderate goodness-of-fit of the GTWR model (adjusted *R*^*2*^ = 0.45) underscore the necessity of further individual-level cohort studies to validate the impacts of interventions.

Some limitations of this study should be acknowledged. First, all GTWR and spatial analyses were conducted at the county aggregate level; thus, ecological fallacy cannot be ruled out. Second, county-level MuCV coverage data were unavailable for inclusion: the MuCV vaccination rate before 2020 cannot be separated from the measles-containing vaccine vaccination rate reports, and the coverage estimate before 2022 relies on low-quality reports of the vaccination rate, making it impossible to adjust for this key confounding factor. Third, the ITS analysis could not disentangle the vaccine policy effect from concurrent COVID-19 NPIs (e.g., masking and school closures), which independently suppressed transmission after 2020. Fourth, passive surveillance is subject to reporting heterogeneity across counties because of variable diagnostic capacity and healthcare-seeking behavior, potentially introducing spatial underreporting bias. Fifth, the GTWR model achieved only a moderate adjusted *R*^*2*^ = 0.45, indicating that more than half of the variance remains unexplained by the selected covariates; unmeasured socioeconomic, behavioral, or environmental factors likely contribute. Sixth, the population mobility metric was derived from a gravity model in the absence of official intercounty migration data and thus serves only as a relative proxy rather than absolute flow count.

## 5 Conclusion

Spatiotemporal, GTWR, and ITS analyses revealed significant nonrandom spatial aggregation of mumps in Hunan (2015–2024), with a persistent hotspot in northeastern Changsha–Zhuzhou. Although the provincial incidence declined after the 2020 two-dose MuCV policy, the clustering intensity increased yearly because of widening disparities, driven by per capita GDP and population mobility intensity in northeastern counties. These heterogeneous effects demonstrate that a regionally differentiated strategy is warranted.

## CRediT authorship contribution statement

**Linlong Liang:** Writing – original draft, Visualization, Software, Methodology, Data curation. **Xiaoyan Liu:** Writing – original draft, Software, Methodology, Funding acquisition. **Fuqiang Liu:** Writing – review & editing, Supervision, Methodology, Data curation. **Lin Liu:** Writing – review & editing, Software, Project administration, Methodology.

## Funding

This research was funded by the 10.13039/501100004735Natural Science Foundation of Hunan Province (grant number: 2025JJ80557).

## Declaration of competing interest

The authors declare that they have no known competing financial interests or personal relationships that could have appeared to influence the work reported in this paper.

## Data Availability

The authors do not have permission to share data.

## Appendix A Supplementary data

Supplementary statistical results for spatiotemporal clustering, socioeconomic drivers and policy evaluation of mumps in Hunan Province, China, 2015–2024. This file contains Supplementary Tables S1–S6.
