# Supplementary material for: Spatiotemporal clustering, socioeconomic drivers and policy evaluation of mumps in Hunan Province, China, 2015–2024
Source: Prev Med Rep. 2026 Aug 17;70:103601. doi: 10.1016/j.pmedr.2026.103601 (PMC13519628; doi:10.1016/j.pmedr.2026.103601)
Supplement: Supplementary file 1 [file mmc1.docx]

**Table S1**

Global spatial autocorrelation analysis of mumps in Hunan Province, 2015 to 2024

| Year | Moran’s *I* | Z Score | *P* Value |
| --- | --- | --- | --- |
| 2015 | 0.32 | 6.06 | ＜0.01 |
| 2016 | 0.20 | 3.99 | ＜0.01 |
| 2017 | 0.24 | 4.63 | ＜0.01 |
| 2018 | 0.20 | 4.05 | ＜0.01 |
| 2019 | 0.55 | 10.47 | ＜0.01 |
| 2020 | 0.55 | 10.50 | ＜0.01 |
| 2021 | 0.55 | 10.41 | ＜0.01 |
| 2022 | 0.54 | 10.27 | ＜0.01 |
| 2023 | 0.61 | 11.60 | ＜0.01 |
| 2024 | 0.67 | 12.63 | ＜0.01 |

Note: *P* values are based on the Z-score derived from the normal approximation for the global Moran’s *I* test.

**Table S2**

Sensitivity analysis of spatiotemporal scan statistics for the mumps outbreak areas in Hunan Province under different spatial window limits (with the time window fixed at 50%)

| Cluster type | Statistical results | Spatial window maximum population limit (%) | | |
| --- | --- | --- | --- | --- |
|  |  | 10 | 20 | 30 |
| Most likely | Time frame | 2017/3/1 to 2019/12/31 | 2016/11/1 to 2019/12/31 | 2016/11/1 to 2019/12/31 |
|  | *LLR* | 24109.12 | 28494.13 | 28494.13 |
|  | *P* value | <0.01 | <0.01 | <0.01 |
| Secondary | Time frame | 2016/11/1 to 2017/6/30 | 2016/11/1 to 2018/7/31 | 2016/11/1 to 2018/7/31 |
|  | *LLR* | 6106.98 | 6801.23 | 7407.34 |
|  | *P* value | <0.01 | <0.01 | <0.01 |
| 2nd secondary | Time frame | 2017/12/1 to 2018/7/31 | 2018/10/1 to 2019/7/31 | 2017/4/1 to 2018/7/31 |
|  | *LLR* | 4628.39 | 2818.94 | 2197.01 |
|  | *P* value | <0.01 | <0.01 | <0.01 |
| 3rd secondary | Time frame | 2017/4/1 to 2018/1/31 | 2017/4/1 to 2018/7/31 | — |
|  | *LLR* | 2923.30 | 2197.01 | — |
|  | *P* value | <0.01 | <0.01 | — |
| 4th secondary | Time frame | 2018/10/1 to 2019/7/31 | 2017/4/1 to 2018/1/31 | — |
|  | *LLR* | 2818.94 | 1767.67 | — |
|  | *P* value | <0.01 | <0.01 | — |
| 5th secondary | Time frame | 2017/11/1 to 2018/7/31 | 2018/12/1 to 2019/7/31 | — |
|  | *LLR* | 2287.03 | 1323.54 | — |
|  | *P* value | <0.01 | <0.01 | — |
| 6th secondary | Time frame | 2018/5/1 to 2018/6/30 | — | — |
|  | *LLR* | 2179.90 | — | — |
|  | *P* value | <0.01 | — | — |
| 7th secondary | Time frame | 2018/11/1 to 2019/7/31 | — | — |
|  | *LLR* | 1511.68 | — | — |
|  | *P* value | <0.01 | — | — |
| 8th secondary | Time frame | 2017/5/1 to 2017/7/31 | — | — |
|  | *LLR* | 1254.84 | — | — |
|  | *P* value | <0.01 | — | — |
| 9th secondary | Time frame | 2015/6/1 to 2016/1/31 | — | — |
|  | *LLR* | 765.78 | — | — |
|  | *P* value | <0.01 | — | — |
| 10th secondary | Time frame | 2017/11/1 to 2018/7/31 | — | — |
|  | *LLR* | 537.74 | — | — |
|  | *P* value | <0.01 | — | — |
| 11th secondary | Time frame | 2015/12/1 to 2017/1/31 | — | — |
|  | *LLR* | 213.99 | — | — |
|  | *P* value | <0.01 | — | — |

Note: Spatial window limits represent the maximum proportion of the total study population that a single cluster could contain; the temporal window was fixed at 50% of the total study period for all three models. *LLR* = Log-likelihood ratio. *P* values were obtained from Monte Carlo simulations (999 replications) using the *LLR* as the test statistic, with the spatial window limit varying as shown.

**Table S3**

Comparison of county numbers within spatiotemporal clusters under 20% and 30% spatial window maximum population limits

| Cluster type | Spatial window maximum population limit (%) | |
| --- | --- | --- |
|  | 20 | 30 |
| Most likely | (12) 12 | (12) 12 |
| Secondary | (28) 29 | (28) 32 |
| 2nd secondary | (25) 38 | (25) 26 |
| 3rd secondary |  | — |
| 4th secondary |  | — |
| 5th secondary |  | — |

Note: Values inside parentheses represent the number of shared county units detected by both the 20% and 30% spatial window models; values outside parentheses denote the total unique counties contained in the cluster under the corresponding spatial threshold. “—” indicates that no independent cluster of this rank was identified under the 30% spatial window model.

**Table S4**

Spatiotemporal clustering of mumps in Hunan Province for 2015 to 2024

| Cluster type | Time frame | Radius (km) | Cluster areas (n) | Number of cases | Expected cases | *LLR* | *RR* | *P* value |
| --- | --- | --- | --- | --- | --- | --- | --- | --- |
| Most likely | 2016/11/1 to 2019/12/31 | (27.93 N, 113.14 E)/43.78 | 12 | 28551 | 4922.2 | 28494.13 | 6.86 | <0.01 |
| Secondary | 2016/11/1 to 2018/7/31 | (28.01 N, 109.51 E)/201.08 | 31 | 17312 | 6029.9 | 7407.34 | 3.10 | <0.01 |
| 2nd secondary | 2017/4/1 to 2018/7/31 | (26.21 N, 113.18 E)/127.67 | 26 | 8677 | 3932.1 | 2197.01 | 2.28 | <0.01 |

Note: *LLR* = Log-likelihood ratio; *RR* = Relative risk. *P* values are derived from the *LLR* statistic using Monte Carlo simulations (999 replications).

**Table S5**

Comparison of the evaluation index results of various models

| Evaluation index | OLS | GWR | GTWR |
| --- | --- | --- | --- |
| *AIC_C_* | 3418.35 | 3303.72 | 2946.90 |
| *R^2^* | 0.04 | 0.20 | 0.46 |
| *AdjR^2^* | 0.04 | 0.20 | 0.45 |
| *RSS* | 1167.26 | 976.46 | 644.09 |

Note: *AIC_C_ =* Akaike Information Criterion; OLS = Ordinary Least Squares; GWR= Geographically Weighted Regression; GTWR = Geographically and Temporally Weighted Regression; *RSS* = Residual Squares.

**Table S6**

The mean regression coefficients of the geographically and temporally weighted regression model

| Independent variable | Min | Lower quartile | Mean | Upper quartile | Max |
| --- | --- | --- | --- | --- | --- |
| Intercept | -1.77 | -0.42 | -0.06 | 0.30 | 1.55 |
| Per Capita GDP | -1.26 | 0.03 | 0.20 | 0.39 | 0.91 |
| Density of medical institutions | -0.26 | -0.05 | 0.02 | 0.06 | 0.52 |
| School density | -0.31 | -0.06 | 0.15 | 0.13 | 3.99 |
| Population mobility intensity | -7.10 | -0.20 | -0.18 | 0.06 | 0.73 |
